# 1,4-Bis(chloro­meth­yl)naphthalene

**DOI:** 10.1107/S1600536808022757

**Published:** 2008-07-26

**Authors:** Muhammad Ilyas Tariq, M. Nawaz Tahir, Ishtiaq Hussain, Ayesha Roohi

**Affiliations:** a Department of Chemistry, University of Sargodha, Sargodha, Pakistan; b Department of Physics, University of Sargodha, Sargodha, Pakistan; c Institute of Chemistry, University of the Punjab, Lahore 54590, Pakistan

## Abstract

In the title mol­ecule, C_12_H_10_Cl_2_, the torsion angles C_r_—C_r_—C_m_—Cl around the C_m_—C_r_ bonds have values of −104.1 (4) and −101.9 (4)°, where C_m_ is a methylene and C_r_ is a ring C atom. The mol­ecules related by translation along the *b* axis are arranged into stacks by π–π inter­actions between unsubstituted and substituted aromatic rings of the naphthalene ring system (centroid–centroid distance = 3.940 Å).

## Related literature

For related literature, see: Basaran *et al.* (1992 ▶); Gabe & Glusker (1971 ▶); Garriz *et al.* (2004 ▶); Ikeda *et al.* (1987 ▶); Kaza­kov (2003 ▶); Li *et al.* (2004 ▶); Mitchell & Iyer (1989 ▶); Tariq *et al.* (2008 ▶); Zhang *et al.* (1989 ▶, 2007 ▶).
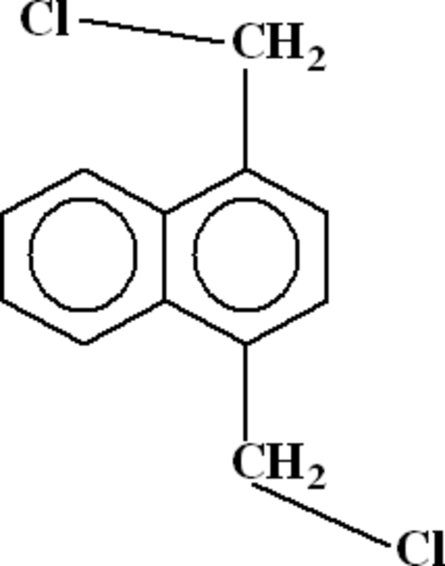


## Experimental

### 

#### Crystal data

C_12_H_10_Cl_2_
*M* *_r_* = 225.10
Monoclinic, 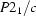

*a* = 13.6887 (11) Å
*b* = 4.5835 (3) Å
*c* = 17.8278 (13) Å
β = 109.666 (4)°
*V* = 1053.31 (13) Å^3^
*Z* = 4
Mo *K*α radiation
μ = 0.57 mm^−1^
*T* = 296 (2) K
0.25 × 0.08 × 0.04 mm

#### Data collection

Bruker Kappa APEX2 diffractometer
Absorption correction: multi-scan (*SADABS*; Bruker, 2005 ▶) *T*_min_ = 0.943, *T*_max_ = 0.974
10310 measured reflections
2073 independent reflections
1220 reflections with *I* > 2σ(*I*)
*R*_int_ = 0.049

#### Refinement

*R*[*F* ^2^ > 2σ(*F* ^2^)] = 0.053
*wR*(*F* ^2^) = 0.167
*S* = 1.05
2073 reflections
127 parameters
H-atom parameters constrained
Δρ_max_ = 0.79 e Å^−3^
Δρ_min_ = −0.28 e Å^−3^

### 

Data collection: *APEX2* (Bruker, 2007 ▶); cell refinement: *APEX2*; data reduction: *SAINT* (Bruker, 2007 ▶); program(s) used to solve structure: *SHELXS97* (Sheldrick, 2008 ▶); program(s) used to refine structure: *SHELXL97* (Sheldrick, 2008 ▶); molecular graphics: *ORTEP-3 for Windows* (Farrugia, 1997 ▶) and *PLATON* (Spek, 2003 ▶); software used to prepare material for publication: *WinGX* (Farrugia, 1999 ▶) and *PLATON*.

## Supplementary Material

Crystal structure: contains datablocks global, I. DOI: 10.1107/S1600536808022757/gk2159sup1.cif

Structure factors: contains datablocks I. DOI: 10.1107/S1600536808022757/gk2159Isup2.hkl

Additional supplementary materials:  crystallographic information; 3D view; checkCIF report

## Figures and Tables

**Fig. 1.**
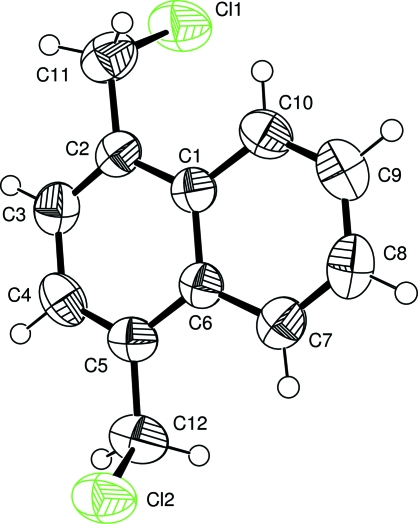
The ORTEP diagram of the title compound with displacement ellipsoids at the 50% probability level. H-atoms are shown by small circles of arbitrary radii.

**Fig. 2.**
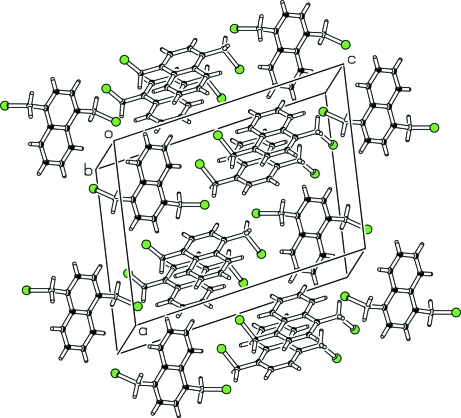
The crystal packing diagram.

## supplementary crystallographic information

### Comment

Naphthalene acetic acid (NAA) is well known growth regulator/stimulator for different varieties of fruits and vegetables (Garriz *et al.*, 2004; Li *et al.*, 2004). Its synthesis has developed a great interest among the chemists and several methods have been reported. One of them is chloromethylation of naphthalene using methylene chloride in the presence of catalysts (Kazakov, 2003; Mitchell & Iyer, 1989; Zhang *et al.*, 1989). During the synthesis of NAA, using formaline and a mixture of acids as a source of insertion of methylene group (Ikeda *et al.*, 1987; Tariq *et al.*, 2008), the title compound  has been isolated.

The crystal structures of 1,4-bis(bromomethyl)benzene (Zhang *et al.*, 2007), 1,4-bis(chloromethyl)benzene (Basaran *et al.*, 1992) and 9,10-bis(chloromethyl)anthracene (Gabe & Glusker, 1971) were published but no analogous derivatives of naphthalene have been reported.

The bond lengths in the naphthalene system are in the range of 1.344 (6)–1.425 (5) Å.  The Cl atoms deviate in opposite directions from the plane of the naphthalene ring by 1.660 (6) Å and 1.559 (6) Å. The closest contacts of Cl atoms with neighbouring molecules are: 3.491 (5) Å  for Cl1···C9^i^ and 3.5581 (16) Å for Cl2···Cl2^ii^ [symmetry codes: (i) -*x*, 1 - *y*, -*z;* (ii) 1 - *x*, 1 - *y*, 1 - *z*].

### Experimental

A mixture of naphthalene (40.0 g), paraformaldehyde (35.0 g), glacial acetic acid (82.0 ml), H_3_PO_4_ (52.0 ml) and concentrated HCl (114.0 ml) was heated in a water bath at 358 K with vigorous stirring for 2 h. Thereafter, the mixture was cooled to room temperature. A solid product was obtained and isolated. It was thoroughly washed with water, ether and n-hexane, respectively in order to remove unreacted material. The product was further purified in hot methanol. Needle-shaped colorless crystals (m.p. 394-396 K) were obtained by recrystallization from ethyl acetate.

### Refinement

H atoms were positioned geometrically, with C—H = 0.93 and 0.97 Å for aromatic and methylene C-atoms, respectively, and constrained to ride on their parent atoms with U_iso_(H)=1.2U_eq_(C).

### Crystal data

**Table:**

C_12_H_10_Cl_2_	*F*_000_ = 464
*M**_r_* = 225.10	*D*_x_ = 1.419 Mg m^−^^3^
Monoclinic, *P*2_1_/*c*	Mo *K*α radiation λ = 0.71073 Å
Hall symbol: -P 2ybc	Cell parameters from 2073 reflections
*a* = 13.6887 (11) Å	θ = 1.6–26.0º
*b* = 4.5835 (3) Å	µ = 0.57 mm^−^^1^
*c* = 17.8278 (13) Å	*T* = 296 (2) K
β = 109.666 (4)º	Needle, colourless
*V* = 1053.31 (13) Å^3^	0.25 × 0.08 × 0.04 mm
*Z* = 4	

### Data collection

**Table:**

Bruker Kappa APEX2 diffractometer	2073 independent reflections
Radiation source: fine-focus sealed tube	1220 reflections with *I* > 2σ(*I*)
Monochromator: graphite	*R*_int_ = 0.049
Detector resolution: 7.40 pixels mm^-1^	θ_max_ = 26.0º
*T* = 296(2) K	θ_min_ = 1.6º
ω scans	*h* = −16→16
Absorption correction: multi-scan(SADABS; Bruker, 2005)	*k* = −5→5
*T*_min_ = 0.943, *T*_max_ = 0.974	*l* = −21→21
10310 measured reflections	

### Refinement

**Table:**

Refinement on *F*^2^	Secondary atom site location: difference Fourier map
Least-squares matrix: full	Hydrogen site location: inferred from neighbouring sites
*R*[*F*^2^ > 2σ(*F*^2^)] = 0.053	H-atom parameters constrained
*wR*(*F*^2^) = 0.167	*w* = 1/[σ^2^(*F*_o_^2^) + (0.0763*P*)^2^ + 0.5487*P*] where *P* = (*F*_o_^2^ + 2*F*_c_^2^)/3
*S* = 1.05	(Δ/σ)_max_ < 0.001
2073 reflections	Δρ_max_ = 0.79 e Å^−^^3^
127 parameters	Δρ_min_ = −0.28 e Å^−^^3^
Primary atom site location: structure-invariant direct methods	Extinction correction: none

### Special details

**Table:**

Geometry. Bond distances, angles etc. have been calculated using the rounded fractional coordinates. All su's are estimated from the variances of the (full) variance-covariance matrix. The cell esds are taken into account in the estimation of distances, angles and torsion angles
Refinement. Refinement of *F*^2^ against ALL reflections. The weighted *R*-factor *wR* and goodness of fit *S* are based on *F*^2^, conventional *R*-factors *R* are based on *F*, with *F* set to zero for negative *F*^2^. The threshold expression of *F*^2^ > σ(*F*^2^) is used only for calculating *R*-factors(gt) *etc*. and is not relevant to the choice of reflections for refinement. *R*-factors based on *F*^2^ are statistically about twice as large as those based on *F*, and *R*- factors based on ALL data will be even larger.

### Fractional atomic coordinates and isotropic or equivalent isotropic displacement parameters (Å^2^)

**Table:**

	*x*	*y*	*z*	*U*_iso_*/*U*_eq_	
Cl1	0.15383 (10)	0.5096 (3)	−0.06255 (6)	0.0825 (5)	
Cl2	0.41801 (9)	0.6216 (3)	0.40488 (6)	0.0694 (4)	
C1	0.2211 (3)	0.3836 (7)	0.1364 (2)	0.0438 (11)	
C2	0.2846 (3)	0.4596 (8)	0.0908 (2)	0.0479 (12)	
C3	0.3638 (3)	0.6492 (9)	0.1214 (2)	0.0566 (14)	
C4	0.3843 (3)	0.7720 (8)	0.1975 (3)	0.0572 (14)	
C5	0.3249 (3)	0.7058 (7)	0.2432 (2)	0.0472 (11)	
C6	0.2416 (3)	0.5067 (7)	0.2133 (2)	0.0428 (11)	
C7	0.1764 (3)	0.4271 (8)	0.2572 (2)	0.0537 (12)	
C8	0.0976 (3)	0.2349 (10)	0.2267 (3)	0.0635 (16)	
C9	0.0776 (3)	0.1120 (9)	0.1520 (3)	0.0642 (16)	
C10	0.1366 (3)	0.1849 (8)	0.1080 (2)	0.0546 (12)	
C11	0.2650 (3)	0.3370 (10)	0.0095 (2)	0.0681 (17)	
C12	0.3470 (3)	0.8534 (9)	0.3220 (2)	0.0631 (16)	
H3	0.40549	0.69924	0.09161	0.0680*	
H4	0.43949	0.90101	0.21711	0.0681*	
H7	0.18769	0.50745	0.30737	0.0642*	
H8	0.05609	0.18432	0.25656	0.0759*	
H9	0.02356	−0.02056	0.13242	0.0771*	
H10	0.12179	0.10348	0.05762	0.0655*	
H11A	0.25290	0.12868	0.01017	0.0818*	
H11B	0.32575	0.36693	−0.00605	0.0818*	
H12A	0.28193	0.91087	0.32822	0.0755*	
H12B	0.38692	1.02904	0.32298	0.0755*	

### Atomic displacement parameters (Å^2^)

**Table:**

	*U*^11^	*U*^22^	*U*^33^	*U*^12^	*U*^13^	*U*^23^
Cl1	0.0917 (9)	0.0956 (9)	0.0466 (7)	0.0066 (7)	0.0055 (6)	0.0041 (6)
Cl2	0.0783 (8)	0.0692 (7)	0.0492 (6)	0.0150 (5)	0.0065 (5)	0.0007 (5)
C1	0.043 (2)	0.0421 (18)	0.042 (2)	0.0109 (16)	0.0085 (16)	0.0062 (16)
C2	0.050 (2)	0.050 (2)	0.043 (2)	0.0115 (18)	0.0149 (17)	0.0073 (17)
C3	0.053 (2)	0.064 (2)	0.055 (3)	0.005 (2)	0.021 (2)	0.015 (2)
C4	0.048 (2)	0.049 (2)	0.064 (3)	−0.0019 (18)	0.005 (2)	0.010 (2)
C5	0.053 (2)	0.0411 (19)	0.041 (2)	0.0119 (17)	0.0071 (18)	0.0081 (16)
C6	0.041 (2)	0.0400 (18)	0.044 (2)	0.0095 (16)	0.0097 (16)	0.0065 (16)
C7	0.052 (2)	0.058 (2)	0.051 (2)	0.0138 (19)	0.0173 (19)	0.0106 (18)
C8	0.051 (2)	0.069 (3)	0.075 (3)	0.009 (2)	0.027 (2)	0.020 (2)
C9	0.054 (3)	0.061 (2)	0.070 (3)	−0.005 (2)	0.011 (2)	0.011 (2)
C10	0.056 (2)	0.051 (2)	0.049 (2)	0.0012 (18)	0.0074 (19)	0.0033 (18)
C11	0.079 (3)	0.072 (3)	0.055 (3)	0.014 (2)	0.025 (2)	0.004 (2)
C12	0.072 (3)	0.049 (2)	0.057 (3)	0.010 (2)	0.007 (2)	−0.0009 (19)

### Geometric parameters (Å, °)

**Table:**

Cl1—C11	1.810 (4)	C8—C9	1.386 (7)
Cl2—C12	1.814 (4)	C9—C10	1.344 (6)
C1—C2	1.419 (6)	C3—H3	0.9300
C1—C6	1.421 (5)	C4—H4	0.9300
C1—C10	1.425 (5)	C7—H7	0.9300
C2—C3	1.353 (6)	C8—H8	0.9300
C2—C11	1.492 (5)	C9—H9	0.9300
C3—C4	1.407 (6)	C10—H10	0.9300
C4—C5	1.365 (6)	C11—H11A	0.9700
C5—C6	1.417 (5)	C11—H11B	0.9700
C5—C12	1.496 (5)	C12—H12A	0.9700
C6—C7	1.419 (6)	C12—H12B	0.9700
C7—C8	1.357 (6)		
			
Cl1···C10	3.464 (4)	C10···H11A	2.7400
Cl1···C9^i^	3.491 (5)	C11···H10	2.6200
Cl2···Cl2^ii^	3.5581 (16)	C12···H7	2.6400
Cl2···C7	3.578 (4)	H3···H11B	2.2900
Cl1···H12A^iii^	3.0500	H3···Cl2^viii^	3.0800
Cl1···H10	2.9800	H4···H12B	2.3100
Cl2···H12B^iv^	3.0400	H4···C4^viii^	2.9200
Cl2···H3^v^	3.0800	H7···Cl2	3.0900
Cl2···H7	3.0900	H7···C12	2.6400
C1···C4^iv^	3.521 (5)	H7···H12A	2.2100
C4···C1^vi^	3.521 (5)	H8···C8^ix^	3.0300
C6···C9^vi^	3.504 (6)	H10···Cl1	2.9800
C6···C12^iv^	3.595 (5)	H10···C11	2.6200
C7···Cl2	3.578 (4)	H10···H11A	2.2300
C7···C12^iv^	3.448 (6)	H11A···C3^iv^	3.0100
C9···C6^iv^	3.504 (6)	H11A···C10	2.7400
C9···Cl1^i^	3.491 (5)	H11A···H10	2.2300
C10···Cl1	3.464 (4)	H11B···H3	2.2900
C12···C7^vi^	3.448 (6)	H12A···C7	2.7200
C12···C6^vi^	3.595 (5)	H12A···C7^vi^	2.8400
C3···H11A^vi^	3.0100	H12A···C8^vi^	2.9600
C4···H4^v^	2.9200	H12A···H7	2.2100
C7···H12A	2.7200	H12A···Cl1^x^	3.0500
C7···H12A^iv^	2.8400	H12B···Cl2^vi^	3.0400
C8···H8^vii^	3.0300	H12B···H4	2.3100
C8···H12A^iv^	2.9600		
			
C2—C1—C6	119.7 (3)	C4—C3—H3	119.00
C2—C1—C10	122.3 (3)	C3—C4—H4	119.00
C6—C1—C10	118.0 (3)	C5—C4—H4	119.00
C1—C2—C3	119.3 (3)	C6—C7—H7	120.00
C1—C2—C11	121.3 (3)	C8—C7—H7	120.00
C3—C2—C11	119.4 (4)	C7—C8—H8	119.00
C2—C3—C4	121.2 (4)	C9—C8—H8	119.00
C3—C4—C5	121.4 (4)	C8—C9—H9	120.00
C4—C5—C6	119.0 (3)	C10—C9—H9	120.00
C4—C5—C12	119.2 (4)	C1—C10—H10	119.00
C6—C5—C12	121.8 (4)	C9—C10—H10	119.00
C1—C6—C5	119.4 (4)	Cl1—C11—H11A	109.00
C1—C6—C7	118.3 (3)	Cl1—C11—H11B	109.00
C5—C6—C7	122.3 (3)	C2—C11—H11A	110.00
C6—C7—C8	120.6 (3)	C2—C11—H11B	109.00
C7—C8—C9	121.5 (4)	H11A—C11—H11B	108.00
C8—C9—C10	119.8 (4)	Cl2—C12—H12A	109.00
C1—C10—C9	121.8 (3)	Cl2—C12—H12B	109.00
Cl1—C11—C2	111.0 (3)	C5—C12—H12A	109.00
Cl2—C12—C5	112.6 (3)	C5—C12—H12B	109.00
C2—C3—H3	119.00	H12A—C12—H12B	108.00
			
C6—C1—C2—C3	−0.1 (5)	C2—C3—C4—C5	−0.3 (6)
C6—C1—C2—C11	−179.5 (3)	C3—C4—C5—C6	0.7 (6)
C10—C1—C2—C3	−179.8 (4)	C3—C4—C5—C12	−177.1 (4)
C10—C1—C2—C11	0.8 (6)	C4—C5—C6—C1	−0.7 (5)
C2—C1—C6—C5	0.5 (5)	C4—C5—C6—C7	−180.0 (4)
C2—C1—C6—C7	179.8 (3)	C12—C5—C6—C1	177.0 (3)
C10—C1—C6—C5	−179.8 (3)	C12—C5—C6—C7	−2.3 (5)
C10—C1—C6—C7	−0.5 (5)	C4—C5—C12—Cl2	−101.9 (4)
C2—C1—C10—C9	179.3 (4)	C6—C5—C12—Cl2	80.4 (4)
C6—C1—C10—C9	−0.4 (6)	C1—C6—C7—C8	1.0 (6)
C1—C2—C3—C4	0.0 (6)	C5—C6—C7—C8	−179.8 (4)
C11—C2—C3—C4	179.4 (4)	C6—C7—C8—C9	−0.5 (6)
C1—C2—C11—Cl1	75.3 (4)	C7—C8—C9—C10	−0.5 (7)
C3—C2—C11—Cl1	−104.1 (4)	C8—C9—C10—C1	1.0 (6)

Symmetry codes: (i) −*x*, −*y*+1, −*z*; (ii) −*x*+1, −*y*+1, −*z*+1; (iii) *x*, −*y*+3/2, *z*−1/2; (iv) *x*, *y*−1, *z*; (v) −*x*+1, *y*−1/2, −*z*+1/2; (vi) *x*, *y*+1, *z*; (vii) −*x*, *y*+1/2, −*z*+1/2; (viii) −*x*+1, *y*+1/2, −*z*+1/2; (ix) −*x*, *y*−1/2, −*z*+1/2; (x) *x*, −*y*+3/2, *z*+1/2.
